# Antioxidant Enzymes Genetic Variants Associated with Urticaria/Angioedema Induced by Cross-Reactive Hypersensitivity to Nonsteroidal Anti-Inflammatory Drugs

**DOI:** 10.3390/ph19040522

**Published:** 2026-03-24

**Authors:** Isabel M. Jiménez-Sánchez, Raquel Jurado-Escobar, José Triano-Cornejo, Rocío Sáenz de Santa María, Rafael Núñez, Imane Allali-Bouamara, Victoria Raya-López, Pedro Chacón, José J. Laguna, María J. Torres, Inmaculada Doña, José A. Cornejo-García

**Affiliations:** 1 Allergy Research Group, IBIMA-Plataforma BIONAND, Instituto de Investigación Biomédica de Málaga, 29009 Malaga, Spain; isabelma1995@gmail.com (I.M.J.-S.); raqueljuradoescobar@gmail.com (R.J.-E.); trianocornejojose@gmail.com (J.T.-C.); rociossm.93@gmail.com (R.S.d.S.M.); imane.all.bmr@gmail.com (I.A.-B.); victoriasofiaraya@gmail.com (V.R.-L.); mjtorresj@gmail.com (M.J.T.); inmadd@hotmail.com (I.D.); 2 Department of Medicine and Dermatology, Faculty of Medicine, University of Malaga, 29010 Malaga, Spain; 3 Allergy Unit, Malaga Regional University Hospital, 29009 Malaga, Spain; 4 Laboratorio de Inmunología y Alergia-Fundación para la Gestión de la Investigación en Salud de Sevilla, Unidad de Gestión Clínica de Alergología, Hospital Universitario Virgen Macarena, 41009 Seville, Spain; pechafe@gmail.com; 5 Departamento de Ciencias de la Salud y Biomédicas, Universidad Loyola Andalucía, 41704 Seville, Spain; 6 Allergy Unit, Cruz Roja Central Hospital, 28003 Madrid, Spain; josejuliolaguna@gmail.com; 7 Faculty of Medicine, Alfonso X El Sabio University, 28691 Madrid, Spain; 8 Inflammatory Diseases Network (RICORS, RD24/0007/0024), Instituto de Salud Carlos III, 28029 Madrid, Spain; 9 Nanostructures for Diagnosing and Treatment of Allergic Diseases Laboratory, Andalusian Centre for Nanomedicine and Biotechnology, IBIMA-Plataforma BIONAND, 29590 Malaga, Spain

**Keywords:** nonsteroidal anti-inflammatory drugs, hypersensitivity, urticaria, antioxidant enzymes, polymorphisms

## Abstract

**Background/Objectives**: Nonsteroidal anti-inflammatory drugs (NSAIDs) are among the most consumed drugs worldwide and the main cause of drug hypersensitivity reactions (HSRs). The most common NSAID-HSR class is cross-hypersensitivity (CR), with patients reacting to NSAIDs from different chemical groups without specific immunological recognition, with NSAID-induced acute urticaria/angioedema (NIUA) being the most frequent clinical phenotype. Although CR-HSRs are triggered by arachidonic acid (AA) alterations following cyclooxygenase (COX)-1 inhibition and cysteinyl-leukotrienes synthesis by 5-lypoxygenase (5-LO), current evidence supports the participation of additional mechanisms. As COX-1 and 5-LO head oxidative pathways, it is conceivable that enzymes participating in antioxidant control are involved in these mechanisms. In addition, as the CR-HSR susceptibility seems to be influenced by genetic factors, the possibility of genetic variants playing a role in such enzymes should not be excluded. **Methods:** In this observational case–control study, we analysed for the first time in NIUA the overall genetic variability in key antioxidant defence enzymes genes, including catalase (*CAT*), glutathione peroxidase (*GPX*)-*1* and *3*, and superoxide dismutase (*SOD*)*-1*. We selected a set of tagging single nucleotide polymorphisms (tSNPs) in these genes using data from Europeans in the 1000 Genomes Project. Two independent Spanish populations (discovery and replication) of NIUA patients and NSAID-tolerant individuals were included. **Results**: Twenty-six tSNPs were genotyped in the discovery population, with three that were significantly associated with NIUA: rs3448 (GPX-1), rs3792798 (GPX-3), and rs10432782 (SOD-1). They were then genotyped in the replication group, with rs3792798 being protective and rs10432782 being associated with an increased NIUA risk. **Conclusions**: Our results suggest that a role for antioxidant enzyme polymorphisms in NIUA is required. Nevertheless, further research is needed to replicate our findings in other populations and their meaning at the molecular level and to investigate the role of such variants in other CR-HSR-induced phenotypes.

## 1. Introduction

The effectiveness of nonsteroidal anti-inflammatory drugs (NSAIDs) in the management of pain and inflammation [1] has made them one of the most consumed drugs worldwide [2,3]. However, they are also the main cause of hypersensitivity reactions (HSRs) to drugs [4,5,6,7,8]. The NSAID-HSR prevalence in the general population is significant, ranging between 0.6% and 5.7%, with higher values in risk populations (30%) [9,10]. In addition, NSAIDs are responsible for more than 40% of emergency care visits because of the drug-HSR suspicion [11].

According to the European Academy of Allergy and Clinical Immunology (EAACI), NSAID-HSRs may be classified as selective and cross-reactive (S- and CR-HSRs, respectively), with the latter being by far the most frequent type [9]. While the S-HSR patients react to a single NSAID or to several NSAIDs from the same chemical group and tolerate acetylsalicylic acid (ASA), the CR-HSR patients develop reactions to at least two chemically unrelated NSAIDs, including ASA. The CR-HSRs are not immunologically mediated and include, at least, the following phenotypes: (i) NSAIDs-exacerbated respiratory disease (NERD), in patients with underlying rhinitis and/or asthma with or without nasal polyposis; (ii) NSAIDs-exacerbated cutaneous disease (NECD) in patients with underlying CSU; and (iii) NSAIDs-induced urticaria/angioedema (NIUA) in otherwise healthy individuals [9]. NIUA is the most frequent phenotype induced by NSAID-HSRs [12,13] and also the most frequent entity due to drug-HSRs [4].

The inhibition of cyclooxygenase (COX)-1 by NSAIDs switches the metabolism of arachidonic acid (AA) from prostaglandins (PGs) towards the 5-lipoxygenase (5-LO) pathway and the biosynthesis of cysteinyl-leukotrienes (CysLTs; mainly LTE4), eliciting a reaction in susceptible individuals. Although CysLTs are recognised to be crucial in CR-HSRs [14,15,16,17,18,19,20,21], current evidence supports that the underlying mechanisms are not completely elucidated, and the participation of additional, concomitant processes that amplify/modulate these reactions should not be ruled out [22,23,24].

PGE synthase uses reduced glutathione (GSH) in PGE2 synthesis [25], and GSH conjugates to LTA4 to form CysLTs [26], i.e., COX-1 and 5-LO enzymes head oxidative pathways. Consequently, it is conceivable that the redox processes could play a certain role in CR-HSRs. In fact, ASA is known to increase reactive oxygen species formation and reduce GSH levels, and previous results from our group have shown a role for oxidative damage in CR-HSRs as revealed by increased levels of carbonyls and of thiobarbituric acid-reactive substances [27,28].

The CR-HSR susceptibility seems to be under the influence of genetic factors, as it has been indicated by their familial aggregation in some studies and in patients with cutaneous involvement [29,30]. Moreover, a recently published study focusing on NERD patients has evidenced that 20% of first-degree relatives had a history of NSAID intolerance and that 11% developed NERD [31]. In addition, single nucleotide polymorphisms (SNPs) from different molecular pathways, including AA metabolism, have been associated with NERD and NECD [32,33,34] and lately with NIUA [35,36].

Taking into account the potential role of an oxidative imbalance in CR-HSRs and their idiosyncratic nature, it is plausible that variants in the main enzymes that are involved in antioxidant defence could be associated with these reactions. Although there is a lack of studies evaluating the potential role of catalase (CAT), glutathione peroxidase (GPX)-1 and 3, and superoxide dismutase (SOD)-1 in the pathogenesis of CR-HSRs, previous studies have reported alterations in these enzymes in allergic reactions, including those induced by drugs [37,38]. In this observational case–control study, we investigated for the first time the overall variability of *CAT*, *GPX*-*1* and *3*, and *SOD-1* genes in NIUA patients. To that end, we have selected a set of tagging SNPs (tSNPs) and genotyped a population of NIUA patients and a population of NSAID-tolerant subjects as controls. To further validate our initial findings, a second unrelated population of patients and controls has been included. To our knowledge, this approach has not been previously conducted in CR-HSRs. We hope our findings help to unravel their underlying molecular mechanisms and to screen for potential biomarkers for diagnostic purposes.

## 2. Results

### 2.1. Demographic and Clinical Data

A total of 1075 individuals were genotyped, encompassing two unrelated, consecutively recruited populations of NIUA patients and controls. The demographic and clinical data from both populations are depicted in Table 1.

No sex or age differences between the NIUA patients and the controls were observed in these populations (Table 1). The mean age for NIUA and the controls was 40.25 ± 15.1 years and 42.68 ± 13.63 years, respectively, in the discovery population (*p* = 0.073), and 41.57 ± 14.82 years and 42.48 ± 11.68 years for NIUA and controls, respectively, in the replication population (*p* = 0.457) (Table 1). No age differences were detected when comparing the discovery versus the replication population in either the patients (*p* = 0.432) or the controls (*p* = 0.848). Concerning sex, females were more commonly affected, but no differences existed between the patients and the controls in the discovery or in the replication populations (*p* = 0.763 and *p* = 0.754, respectively) (Table 1). In addition, no sex differences were observed in either the patients or the controls when the two populations were compared (*p* = 0.051 and *p* = 0.098, respectively).

We did not find statistically significant differences in the total number of reported reactions when the NIUA patients from the discovery and the replication populations were compared (3.85 ± 1.71 and 3.64 ± 1.58, respectively; *p* = 0.177). Concerning the number of reported reactions to specific pharmacological groups, no differences were detected between the two included populations (*p* = 0.396). The propionic acid derivatives were the most frequently involved in the NIUA populations, followed by ASA and pyrazolones.

### 2.2. Genetic Association Study

Twenty-six tSNPs were selected in *CAT* (n = 12), *GPX*-1 (n = 1), *GPX*-3 (n = 6), and *SOD-1* (n = 7) (Table 2) and genotyped in the discovery population. None of them deviated significantly from the HWE, so they were all retained for association evaluation. Across all individuals, the genotyping call rate was >99% for all tSNPs.

The results from the genetic association study in the discovery population in the dominant, recessive and additive models of inheritance are presented in Table 3.

The rs3448 polymorphism in GPX-1 was associated with a diminished NIUA risk in the dominant (OR = 0.47, 95% CI = 0.33–0.68; *p* = 3.76 × 10^−5^) and the additive (OR = 0.61, 95% CI = 0.45–0.83; *p* = 1.42 × 10^−3^) models. These associations remained statistically significant after the Bonferroni correction (p* = 9.79 × 10^−4^ for the dominant model, and p* = 0.037 for the additive model) (Table 3). Similarly, the rs3792798 variant in GPX-3 was associated with a diminished risk considering the dominant (OR = 0.39, 95% CI = 0.23–0.65; *p* = 1.86 × 10^−4^) and the additive model (OR = 0.47, 95% CI = 0.29–0.75; *p* = 9.16 × 10^−4^). These associations also remained significant after the Bonferroni correction (p* = 4.85 × 10^−3^ for the dominant model, and p* = 0.024 for the additive model) (Table 3). Finally, the rs10432782 tSNP in SOD-1 was associated with an increased NIUA risk in the recessive (OR = 5.59, 95% CI = 2.53–12.35; *p* = 1.56 × 10^−6^) and the additive (OR = 1.6, 95% CI = 1.21–2.13; *p* = 8.74 × 10^−4^) models. This variant remained statistically associated after correction (p* = 4.05 × 10^−5^ for the dominant model, and p* = 0.022 for the additive model) (Table 3).

Although not statistically significant, some trends were observed for rs3805435 in GPX-3 (recessive model, *p* = 0.053), and the SOD-1 variant rs178883442 (dominant model, *p* = 0.08; additive model, *p* = 0.074).

The three nominally associated tSNPs found to be associated with NIUA in the discovery population were further genotyped in the independent case–control replication population and analysed for association after checking that all of them deviated significantly from the HWE in controls (Table 4).

The GPX-1 rs3792798 variant was found to be protective in the replication cohort in the dominant (OR = 0.58, 95% CI = 0.37–0.9; *p* = 0.014) and the additive (OR = 0.61, 95% CI = 0.4–0.92; *p* = 0.015) inheritance models. In addition, it retained a statistical significance after the Bonferroni correction was performed on these models (*p* = 0.043 and *p* = 0.045, respectively). The rs10432782 tSNP in SOD-1 was associated with an increased risk of developing NIUA in the dominant (OR = 1.92, 95% CI = 1.33–2.77; *p* = 5.2 × 10^−4^) and the additive (OR = 1.81, *p* = 1.32–2.49; *p* = 2.05 ×10^−4^) models, with these associations remaining significant after multiple testing corrections (*p* = 1.56 × 10^−3^ and *p* = 6.14 × 10^−4^, respectively). Although this variant was also marginally associated with the recessive model (OR = 2.86, 95% CI = 1.11–7.39; *p* = 0.026), the association did not survive the Bonferroni correction (*p* = 0.079) (Table 4).

### 2.3. Gene Expression and Enzyme Activities

**Figure 1:**
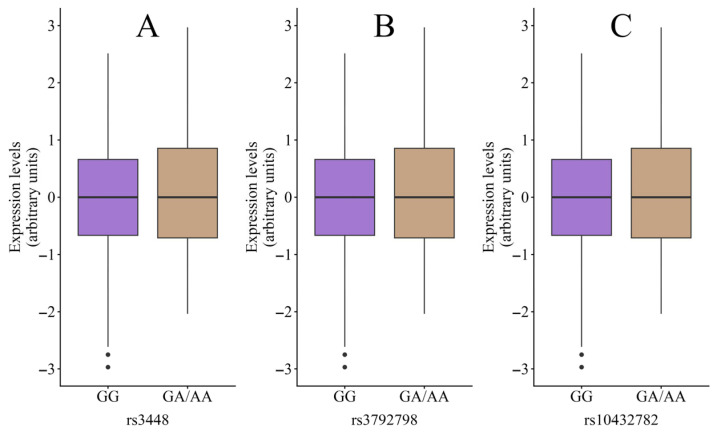
The expression quantitative trait locus (eQTL) analysis for the variants rs3448 (**A**), rs3792798 (**B**) and rs10432782 (**C**) using available data from GTEx.

Concerning the expression quantitative trait locus (eQTL) analysis, the rs3448 variant did not show statistically significant differences between genotypes (CC, n = 354, −0.06 ± 0.09 relative units; CT/TT, n = 316, 0.06 ± 1.04 relative units; *p* = 0.116). No differences were found in either the rs3792798 (GG, n = 532, −0.01 ± 0.97 relative units; GA/AA, n = 138, 0.05 ± 1.06 relative units; *p* = 0.532) or the rs10432782 (TT, n = 498, −0.02 ± 1.03 relative units; TG/GG, n = 172, 0.06 ± 0.88 relative units; *p* = 0.322) polymorphisms (Figure 1).

**Figure 2:**
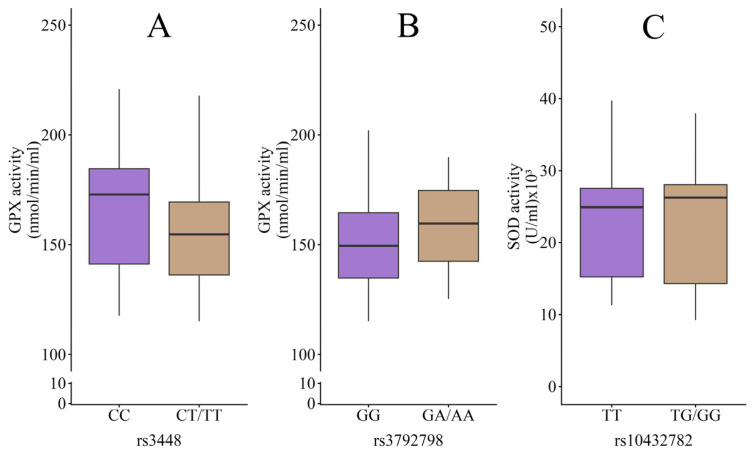
The enzyme activity evaluation in NIUA patients: GPX in the rs3448 (**A**) and rs3792798 variants (**B**), and SOD in rs10437782 (**C**).

When enzyme activities were assessed, no differences were detected between genotypes for GPX neither for rs3448 (CC, n = 15, 152.29 ± 22.01 nmol/min/mL; CT/TT, n = 8, 145.43 ± 23.62 nmol/min/mL; *p* = 0.495) nor for rs3792798 (GG, n = 19, 157.09 ± 24.23 nmol/min/mL; GA/AA, n = 4, 152.11 ± 26.93 nmol/min/mL; *p* = 0.716). Finally, SOD activity did not differ between the rs10432782 genotypes (TT, n = 17, 23.05 ± 8.99 × 103 U/mL; TG/GG, n = 6, 26.45 ± 7.13 × 103 U/mL; *p* = 0.414) (Figure 2).

## 3. Discussion

The high prescription rate and over-the-counter availability of NSAIDs have led them to be among the most used medicines all over the world and emerge as the main triggers of drug-HSRs [4,5,6,7,8]. Although two broad classes of NSAID-HSRs are considered in the EAACI classification, CR-HSRs are the most frequent, with patients reacting to two or more NSAIDs from different chemical groups, including ASA [9,12,13], with NIUA being the most common clinical phenotype [4,12,13].

Alterations in the AA metabolic pathway are accepted to be crucial in the CR-HSRs: COX-1 inhibition by NSAIDs shifts such pathways from PGs to CysLTs biosynthesis, which in some individuals provoke the reactions [9]. However, our understanding of the underlying mechanism is still incomplete, and the concurrence of additional processes in amplifying or modulating CR-HSRs should be contemplated. Considering that COX-1 and 5-LO head oxidative pathways, and our preliminary findings of the potential involvement of oxidative damage [27,28], along with the fact that the CR-HSR susceptibility may be influenced by genetic factors, it is believable that variants in the main antioxidant defence enzymes could play a role.

In this observational case–control study, we have evaluated the overall genetic variability in some of the principal antioxidant enzymes: CAT, GPX-1, GPX-3 and SOD-1. From the 26 initially evaluated tag-SNPs in these genes, a 3’-UTR variant in GPX-1 (rs3448) and two intronic variants (rs3792798 in GPX-3 and rs10432782 in SOD-1) were associated with NIUA in the discovery population and afterwards genotyped in the replication population. Two of them, rs3792798 and rs10432782, remained statistically associated after the Bonferroni correction, whereas rs3448 did not surpass the multiple testing correction. The rs3792798 genetic variant was found to be protective, whereas rs10432782 was associated with an increased NIUA risk.

In general, the available information regarding variants in genes that are included in this study is limited. In fact, there is a lack of studies evaluating the rs3792798 polymorphism, although in the Han Chinese ethnic group, it has been associated with a diminished risk of developing generalised pustular psoriasis, a severe and rare type of psoriasis [39]. Other variants in GPX-3 have been shown to be related to liver homeostasis [40] and to confer protection in a variety of pathological conditions, such as against an asthma onset [41], sudden sensorineural hearing loss [42], as well as differentiated thyroid [43] and gastric cancer [44]. Several studies have found the intronic SOD-1 variant rs10432782 to be associated with an increased risk of aggressive/advanced prostate [45,46] and bladder [47] cancer and an increased risk of death from cardiovascular causes (sudden death, fatal myocardial infarction or stroke) in type 2 diabetic individuals [48]. Of particular interest is amyotrophic lateral sclerosis (ALS) because of its relationship with the variants in SOD-1: over 200 mutations have been reported for this gene in the Human Gene Mutation Database, of which more than 100 are associated with ALS. Some of these variations imply misfolding and protein aggregation, leading to the death of motor neurons [49] and disrupting pre-mRNA splicing [50].

Despite the scarce clinical data involving the intronic variants rs3792798 and rs10432782, it is known that introns are crucial regulators of alternative splicing and gene expression because of their role as transcription factor binding sites and as modulators of transcription and transcription stability [51]. Thus, splicing dysregulation could impact neuromuscular disorders such as ALS [51], cancer [52,53], Alzheimer’s disease [54] and cardiovascular disease [55], among others.

The GPX-1 rs3448 polymorphism, nominally associated with a reduced NIUA risk in the discovery population but only marginally in the replication one, is located in the 3’-UTR. As for the other two variants, clinical information is scant and mainly limited to prostate [46,56] and oestrogen receptor breast cancer [57], as well as kidney complications in patients with type 2 diabetes [58]. However, 3’-UTR variants can modify cis-regulatory elements, modifying mRNA stability, translation efficiency, and subcellular location. Through the interactions with RNA-binding proteins and miRNAs, the 3’-UTR renders mRNA crucial in a great diversity of regulatory networks [59]. In fact, polymorphisms in this region have been associated with a plethora of pathologies, including cancer [60,61,62] and autoimmune diseases [63,64,65], as well as drug response [66,67,68].

The lack of association of the rs3448 variant in the replication population may be due to a decrease in the statistical power, which consequently affected the OR and the *p*-value. Although this polymorphism did not surpass the strict Bonferroni correction, it was marginally associated with a decreased NIUA risk. In addition, it should be noted that, even within the same country, regional genetic background differences may exist due to different factors (historical migrations, isolation, or admixture). Although all included individuals in our study reported Spanish ancestry, subtle ancestry differences should not be excluded as a source of variation in the ORs. Additionally, environmental or lifestyle factors (e.g., diet, smoking, pollution, and socioeconomic status) may interact differently with genetic variants across populations, leading to variations in effect estimates. In addition, the possibility of these variants not being the causal ones by simply tagging them, whether through coding or not, cannot be ruled out. Consequently, if the linkage disequilibrium between the associated tSNP and the true causal variant differs in our populations, the observed OR and *p*-value may also differ. Moreover, these intronic variants could have a role in the cellular processes affecting gene expression, such as variations in the methylation patterns that could modulate drug-HSRs [69,70,71], including those to NSAIDs [72,73].

Our results, together with our preliminary findings on the potential involvement of oxidative damage [27,28], suggest a role for the alterations of antioxidant defence systems in NIUA. However, in addition to these two exploratory studies being performed in a limited number of NIUA patients, DNA was not available for most of them. Consequently, we were not able to evaluate if particular genotypes in associated genetic variants showed a different or a similar oxidative damage profile.

As stated, COX-1 and 5-LO pathways encompass redox reactions, which support that an imbalance in such reactions may have a role in NIUA. However, what are the potential ways that oxidative stress may participate in NIUA central processes, such as mast cells (MCs) degranulation and CysLTs production? The earliest studies demonstrated that MC activation is accompanied by reactive oxygen species production (ROS), which develops a regulatory role in the MC inflammatory mediators’ release, including histamine and CysLTs [74]. Moreover, 5-LO, the key enzyme in CysLTs biosynthesis, makes a crucial contribution to the ROS generation in both human and mouse bone marrow-derived MCs [75]. In antigen-stimulated MCs, superoxide and hydrogen peroxide appear to regulate intracellular and/or plasma membrane Ca2+ channels and related signalling pathways involving different kinases [76,77]. In anaphylaxis, in vitro MC activation increases oxidative stress through NADPH-oxidases, with antioxidant administration reducing the mediators’ release [78]. Similar results were also described in an AA-supplemented rat MC line, with an increase in Ca2+ protein tyrosine phosphorylation [79]. However, whether these specific or other related mechanisms participate in NIUA needs further research.

## 4. Materials and Methods

### 4.1. Subjects

Two consecutively recruited independent populations of unrelated NIUA patients and controls, which we named the discovery and replication populations, were included. The NIUA patients from both populations were aged 18–60 years and had self-reported Spanish ancestry. The discovery population was recruited from January 2018 to September 2020 in the Allergy Unit of the Malaga Regional University Hospital and encompassed 247 NIUA patients and 294 controls. The replication population was recruited between November 2020 and March 2022 in the Allergy Unit of the Cruz Roja Central Hospital (Madrid, Spain) and was composed of 205 NIUA patients and 329 controls (Table 1).

All of the patients reported at least two cutaneous reactions (NIUA) after the intake of NSAIDs from at least two different chemical groups. The patients with CSU were ruled out. In all cases, CR-HSRs were confirmed through a positive ASA drug provocation test (DPT) [80,81,82].

The age- and sex-matched subjects were included as controls. They all declared a regular or occasional intake of NSAIDs without developing a reaction and had no history of drug hypersensitivity or CSU.

The study was approved by the Ethics Committee of the participant centres and carried out following the principles of the Helsinki Declaration. All of the participants gave informed consent.

### 4.2. ASA Drug Provocation Test

The ASA DPT was conducted in a single-blind manner, providing placebo capsules at different intervals during the first day, as described [81]. Both the ASA and the placebo were administered in opaque capsules that were supplied by the hospital pharmacy service. The other drugs were withheld before testing, as recommended [83].

On the second day, two doses of ASA (50 and 100 mg) were given orally with a 3 h interval. If no symptoms appeared, two larger doses (250 and 500 mg) were provided on the third day, with a 3 h interval. The DPT was stopped if the patients developed symptoms or they were being evaluated and treated [81]. On the contrary, the therapeutic dose (500 mg) was given for 2 days in an 8 h interval after a gap of 24 h [81].

### 4.3. Genotyping

The procedure has been described elsewhere [35,36]. Briefly, the data for the individuals with European ancestry were downloaded from phase three of the 1000 Genome Project website (https://www.internationalgenome.org/data-portal/data-collection/phase-3, accessed on 22 March 2021). Tabix 1.10.2 [84] and VCFtools 0.1.16 [85] were used to extract the regions encompassing the *CAT*, *GPX*-1, *GPX-3*, and *SOD-1* genes plus 2 kb upstream and downstream from the gene’s start and end positions, respectively. The obtained files were loaded into Haploview after their conversion [86]. The non-biallelic and indel markers were discarded, and common tSNPs (MAF ≥ 5%) were selected based on their linkage disequilibrium with Tagger [87], using pairwise tagging and an r2 threshold of 0.8.

The genomic DNA was obtained from peripheral blood with the FlexiGene DNA Kit system (QIAGEN, Germantown, MD, USA). The samples from the discovery population were genotyped using the iPlex Sequenom MassArray technology, whereas those from the replication population were genotyped using TaqMan probes (Thermo Fisher Scientific, Waltham, MA, USA) and qPCR (Thermo Fisher Scientific, Waltham, MA, USA). Ten samples from each population were genotyped using both technologies in order to ensure they gave identical results. The agreement was of 100%.

The discovery phase of the study was estimated to use 80% of its power to detect an associated tSNP with α = 0.05, assuming a disease prevalence of 0.01, a risk allele frequency of 0.2 and a relative risk of >1.5 [88].

### 4.4. Gene Expression and Enzyme Activities

The eQTL analysis was performed using data from the GTEx project database [89], accessed via the *gtexr* 0.2.1 R package [90].

The enzyme activities were determined in available cell lysates from a total of 23 NIUA patients from those included in the genetic association study. The GPX activity was measured using the Glutathione Peroxidase Assay Kit, whereas the SOD activity was determined with the Superoxide Dismutase Assay Kit, both from Cayman Chemical (Ann Arbor, MI, USA).

### 4.5. Statistical Analysis

The Mann–Whitney U test was used to compare continuous variables between groups, and the χ2 test was used for categorical ones. The Hardy–Weinberg equilibrium (HWE) and individual tSNP association tests with NIUA were conducted through SNPassoc, considering dominant, recessive, and additive models for all tag-SNPs [91]. The latter was performed using logistic regressions to calculate the tSNP effects as odds ratios with 95% confidence intervals. A *p*-value ≤ 0.05 after the Bonferroni multiple testing correction was considered statistically significant. The Bonferroni correction thresholds were 1.92 × 10^−3^ (0.05/26) for the discovery cohort, and 0.017 (0.05/3) for the replication one. The normality and homoscedasticity of eQTL and enzyme activities were assessed using the Shapiro–Wilk and Levene tests, respectively. The means between these considered groups were compared using Student’s *t*-test.

## 5. Conclusions

To summarise, our results suggest that genetic variants in antioxidant defence enzymes may play a role in the development of NIUA. Some such enzymes have been associated with human diseases and their responses to drugs [92]. In two consecutive, independent populations, we have found two intronic polymorphisms in GPX-3 and SOD-1 to be associated with NIUA; however, the characterisation of the molecular mechanisms supporting such associations needs further investigation.

To our knowledge, this is the first analysis of the overall genetic variability in CAT, GPX-1, GPX-3 and SOD-1 in CR-HSRs. However, our study suffers from some limitations. In fact, we did not find any relationship between the associated variants and the enzyme expressions and activities. Nevertheless, the enzyme gene expression evaluation was performed using a public database, and our enzyme activity study was performed in a limited number of patients. In addition to the gap in these genetic associations at the molecular level, a further evaluation of these variants/genes in other populations and ethnic groups should be performed to determine if our results are applicable or not to populations with ethnicities other than the Spanish. These studies would provide a better comprehension of the genetics of CR-HSRs and their underlying mechanisms and contribute to the characterisation of biomarkers for their use in diagnostics.

## Figures and Tables

**Table 1:** The demographic and clinical data for NIUA patients and NSAID-tolerant controls.

	Discovery Population			Replication Population		
Variable	NIUA (n = 247)	Controls (n = 294)	*p*-Value	NIUA (n = 205)	Controls (n = 329)	*p*-Value
Age, years (mean ± SD) *	40.25 ± 15.1	42.68 ± 13.63	0.073	41.57 ± 14.82	42.48 ± 11.78	0.457
Female counts, n (%) **	148 (59.9)	181 (61.6)	0.763	176 (53.5)	106 (51.7)	0.754
No. of reactions (mean ± SD) ***	3.85 ± 1.71	NA	NA	3.64 ± 1.58	NA	NA
Pharmacological culprit drug, No. of reactions (%) ****		NA	-		NA	-
*Propionic acid derivatives*	422 (44.4)			297 (39.5)		
*Acetylsalicylic acid*	172 (18.1)			156 (20.7)		
*Pyrazolones*	181 (19)			158 (21)		
*Arylacetic acid derivatives*	100 (10.5)			86 (11.4)		
*Others*	76 (8)			55 (7.3)		

Abbreviation: NA, not applicable; NIUA, NSAIDs-induced acute urticaria/angioedema. * No significant age differences were detected when comparing the discovery versus the replication population in either the patients (*p* = 0.432) or the controls (*p* = 0.848). ** No statistically significant sex differences were found in the patients (*p* = 0.098) or the controls when the two included populations were compared (*p* = 0.051). *** No significant differences were found regarding the number of reactions between NIUA patients from the discovery and replication populations (*p* = 0.177). **** No significant differences were found regarding the culprit pharmacological groups involved in the reactions between the discovery and the replication NIUA patient populations (*p* = 0.396). The names of specific NSAIDs groups are indicated using italics typeface.

**Table 2:** The selected tSNPs.

Gene	Tag-SNPs	Position	Alleles (M/m)
*CAT*	rs564250	34437314	C/T
	rs769214	34438170	A/G
	rs1001179	34438684	C/T
	rs769217	34461361	C/T
	rs7104301	34472091	A/G
	rs475043	34472254	T/C
	rs16925614	34470762	C/T
	rs551929	34465645	G/T
	rs11032703	34448109	C/T
	rs525938	34442046	T/C
	rs554576	34462347	A/T
	rs480496	34467282	G/A
*GPX-1*	rs3448	49359318	C/T
*GPX-3*	rs3792798	151022204	G/A
	rs3792796	151022929	C/G
	rs2070593	151028379	G/A
	rs4958872	151022773	T/C
	rs2233311	151030658	C/A
	rs3805435	151021735	T/C
*SOD-1*	rs36233090	31657962	C/G
	rs17881274	31658867	C/T
	rs17880135	31669690	T/G
	rs17883442	31669841	T/C
	rs2833474	31670038	A/G
	rs12626475	31670616	A/G
	rs10432782	31664078	T/G

Abbreviations: *CAT*, catalase; *GPX*, glutathione peroxidase; M, major allele; m, minor allele; *SOD-1*, superoxide dismutase; tSNPs, tagging single nucleotide polymorphisms.

**Table 3:** The genetic association study with the selected tSNPs in the discovery population.

Model										
Gene	tSNPs	Dominant			Recessive			Additive		
		OR (95% CI)	*p*	p*	OR (95% CI)	*p*	p*	OR (95% CI)	*p*	p*
*CAT*	rs564250	0.93 (0.66–1.31)	0.674		1.43 (0.65–3.16)	0.373		1 (0.75–1.33)	0.981	
	rs769214	1.07 (0.76–1.51)	0.686		0.86 (0.51–1.43)	0.552		1 (0.78–1.28)	0.998	
	rs1001179	1.01 (0.71–1.44)	0.959		0.69 (0.27–1.79)	0.445		0.97 (0.71–1.31)	0.835	
	rs769217	0.99 (0.71–1.39)	0.958		0.81 (0.41–1.6)	0.541		0.96 (0.73–1.26)	0.773	
	rs7104301	0.94 (0.67–1.32)	0.718		0.85 (0.47–1.55)	0.589		0.93 (0.72–1.21)	0.605	
	rs475043	1.14 (0.81–1.61)	0.444		1.04 (0.58–1.84)	0.903		1.09 (0.84–1.42)	0.521	
	rs16925614	0.89 (0.61–1.3)	0.544		1.21 (0.34–3.8)	0.744		0.93 (0.66–1.3)	0.659	
	rs551929	1.12 (0.62–2.03)	0.709		1.21 (0.08–19.39)	0.895		1.11 (0.64–1.95)	0.706	
	rs11032703	1.22 (0.78–1.21)	0.552		1.21 (0.17–8.63)	0.852		1.2 (0.79–1.83)	0.391	
	rs525938	1.05 (0.75–1.4)	0.762		0.85 (0.48–1.5)	0.578		1 (0.77–1.29)	0.979	
	rs554576	1.06 (0.74–1.52)	0.742		0.77 (0.48–1.24)	0.278		0.96 (0.75–1.23)	0.729	
	rs480496	0.93 (0.66–1.31)	0.674		1.43 (0.65–3.16)	0.373		1 (0.75–1.33)	0.981	
*GPX-1*	**rs3448**	**0.47** **(0.33–0.68)**	**3.76 ×** **10^−5^**	**9.79 × 10^−4^**	1.32 (0.59–2.95)	0.495		**0.61** **(0.45–0.83)**	**1.42 × 10^−3^**	**0.037**
*GPX-3*	**rs3792798**	**0.39** **(0.23–0.65)**	**1.86 × 10^−4^**	**4.85 × 10^−3^**	1.21 (0.24–6.04)	0.819		**0.47** **(0.29–0.75)**	**9.16 × 10^−4^**	**0.024**
	rs3792796	0.78 (0.55–1.11)	0.169		1.15 (0.72–1.83)	0.561		0.92 (0.72–1.18)	0.516	
	rs2070593	0.9 (0.62–1.32)	0.599		1.84 (0.65–5.24)	0.249		0.98 (0.71–1.36)	0.927	
	rs4958872	0.81 (0.58–1.15)	0.238		1.03 (0.57–1.85)	0.923		0.89 (0.69–1.16)	0.387	
	rs2233311	1.21 (0.76–1.94)	0.422		2.42 (0.22–26.84)	0.456		1.23 (0.79–1.91)	0.365	
	rs3805435	0.85 (0.54–1.57)	0.761		6.13 (0.71–52.82)	0.053		0.97 (0.64–1.45)	0.867	
*SOD-1*	rs36233090	0.92 (0.54–1.57)	0.762		0.6 (0.05–6.66)	0.671		0.91 (0.55–1.5)	0.708	
	rs17881274	0.92 (0.54–1.57)	0.762		1.21 (0.17–8.63)	0.852		0.94 (0.58–1.54)	0.817	
	rs17880135	0.8 (0.44–1.47)	0.476		1.21 (0.08–19.38)	0.895		0.83 (0.47–1.47)	0.52	
	rs17883442	1.69 (0.94–3.06)	0.08		2.43 (0.22–26.95)	0.454		1.64 (0.95–2.84)	0.074	
	rs2833474	0.94 (0.63–1.4)	0.744		0.45 (0.12–1.7)	0.213		0.89 (0.62–1.27)	0.518	
	rs12626475	0.76 (0.54–1.07)	0.117		1.09 (0.67–1.78)	0.729		0.89 (0.69–1.14)	0.344	
	**rs10432782**	**1.42** **(0.97–2.08)**	**0.07**		**5.59** **(2.53–12.35)**	**1.56 × 10^−6^**	**4.05 × 10^−5^**	**1.6** **(1.21–2.13)**	**8.74 × 10^−4^**	**0.022**

Abbreviations: CAT, catalase; GPX, glutathione peroxidase; p*, Bonferroni-corrected *p*-value; SOD, superoxide dismutase. Statistically significant associations after Bonferroni correction are indicated in boldface.

**Table 4:** The genetic association study in the replication population.

tSNPs	Dominant			Recessive			Additive		
	OR (95% CI)	*p*	p*	OR (95% CI)	*p*	p*	OR (95% CI)	*p*	p*
rs3448	0.7 (0.49–1.01)	0.055		0.63 (0.27–1.45)	0.263		0.74 (0.54–1)	0.045	0.131
**rs3792798**	**0.58** **(0.37–0.9)**	**0.014**	**0.043**	0.53 (0.11–2.65)	0.419		**0.61** **(0.4–0.92)**	**0.015**	**0.045**
**rs10432782**	**1.92** **(1.33–2.77)**	**5.2 × 10^−4^**	**1.56 × 10^−3^**	2.86 (1.11–7.39)	0.026	0.079	**1.81** **(1.32–2.49)**	**2.05 × 10^−4^**	**6.14 × 10^−4^**

Abbreviations: p*, Bonferroni-corrected *p*-value. Statistically significant associations after Bonferroni correction are indicated in boldface.

## Data Availability

The original contributions presented in this study are included in the article. Further inquiries can be directed to the corresponding author.

## Abbreviations

The following abbreviations are used in this manuscript:

NSAIDs	Nonsteroidal anti-inflammatory drugs
HSRs	Hypersensitivity reactions
EAACI	European Academy of Allergy and Clinical Immunology
ASA	Acetylsalicylic acid
S-HSRs	Selective HSRs
C-HSRs	Cross-reactive HSRs
NERD	NSAIDs-exacerbated respiratory disease
NECD	NSAIDs-exacerbated cutaneous disease
NIUA	NSAIDs-induced urticaria/angioedema
COX-1	Cyclooxygenase-1
PGs	Prostaglandins
CysLTs	Cysteinyl-leukotrienes
GSH	Reduced glutathione
SNPs	Single nucleotide polymorphisms
CAT	Catalase
GPX	Glutathione peroxidase
SOD	Superoxide dismutase
tSNPs	Tagging SNPs
eQTL	Expression quantitative trait locus
HWE	Hardy–Weinberg equilibrium
