# Correction: Jiménez-Sánchez et al. Antioxidant Enzymes Genetic Variants Associated with Urticaria/Angioedema Induced by Cross-Reactive Hypersensitivity to Nonsteroidal Anti-Inflammatory Drugs. *Pharmaceuticals* 2026, *19*, 522

**DOI:** 10.3390/ph19060911

**Published:** 2026-06-09

**Authors:** Isabel M. Jiménez-Sánchez, Raquel Jurado-Escobar, José Triano-Cornejo, Rocío Sáenz de Santa María, Rafael Núñez, Imane Allali-Bouamara, Victoria Raya-López, Pedro Chacón, José J. Laguna, María J. Torres, Inmaculada Doña, José A. Cornejo-García

**Affiliations:** 1 Allergy Research Group, IBIMA-Plataforma BIONAND, Instituto de Investigación Biomédica de Málaga, 29009 Malaga, Spain; isabelma1995@gmail.com (I.M.J.-S.); raqueljuradoescobar@gmail.com (R.J.-E.); trianocornejojose@gmail.com (J.T.-C.); rociossm.93@gmail.com (R.S.d.S.M.); rafnunser@gmail.com (R.N.); imane.all.bmr@gmail.com (I.A.-B.); victoriasofiaraya@gmail.com (V.R.-L.); mjtorresj@gmail.com (M.J.T.); inmadd@hotmail.com (I.D.); 2 Department of Medicine and Dermatology, Faculty of Medicine, University of Malaga, 29010 Malaga, Spain; 3 Allergy Unit, Malaga Regional University Hospital, 29009 Malaga, Spain; 4 Laboratorio de Inmunología y Alergia-Fundación para la Gestión de la Investigación en Salud de Sevilla, Unidad de Gestión Clínica de Alergología, Hospital Universitario Virgen Macarena, 41009 Seville, Spain; pechafe@gmail.com; 5 Departamento de Ciencias de la Salud y Biomédicas, Universidad Loyola Andalucía, 41704 Seville, Spain; 6 Allergy Unit, Cruz Roja Central Hospital, 28003 Madrid, Spain; josejuliolaguna@gmail.com; 7 Faculty of Medicine, Alfonso X El Sabio University, 28691 Madrid, Spain; 8 Inflammatory Diseases Network (RICORS, RD24/0007/0024), Instituto de Salud Carlos III, 28029 Madrid, Spain; 9 Nanostructures for Diagnosing and Treatment of Allergic Diseases Laboratory, Andalusian Centre for Nanomedicine and Biotechnology, IBIMA-Plataforma BIONAND, 29590 Malaga, Spain

In the original publication [1], there was a mistake in Figure 1 as published. There was a mistake in the plotting of data from the GTEx project database, affecting Figure 1A,C. The corrected Figure 1 appears below. The authors state that the scientific conclusions are unaffected. This correction was approved by the Academic Editor. The original publication has also been updated.

## Figures and Tables

**Figure 1:**
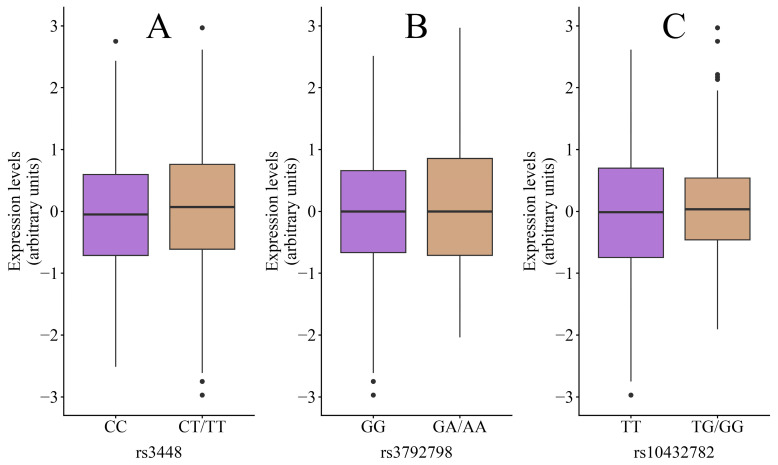
The expression quantitative trait locus (eQTL) analysis for the variants rs3448 (**A**), rs3792798 (**B**) and rs10432782 (**C**) using available data from GTEx.
